# Adolescent Anxiety During the COVID‐19 Pandemic: A Qualitative Systematic Review of Risk and Protective Factors

**DOI:** 10.1002/jad.70038

**Published:** 2025-08-20

**Authors:** Buket Kara, Nitzan Scharf, Kathleen C McCormick, Linda Bhreathnach, Candace Currie, Jennifer Symonds

**Affiliations:** ^1^ Division of Health Research Lancaster University Lancaster UK; ^2^ Department of Human Development and Family Science University of Georgia Athens Georgia USA; ^3^ Georgia Center for Developmental Science University of Georgia Athens Georgia USA; ^4^ Department of Psychology Cornell University Ithaca New York USA; ^5^ Social Research Institute University College London London UK; ^6^ Glasgow Caledonian University London Glasgow UK

**Keywords:** adolescence, anxiety, COVID‐19, protective factors, risk factors, systematic review

## Abstract

**Introduction:**

The COVID‐19 pandemic significantly disrupted adolescents' lives, leading to increased stress and anxiety rates globally. Although existing research highlights the necessity of understanding the increased rates of anxiety in adolescents during and after the pandemic, it offers little insight into the risk and protective factors for the development of adolescent anxiety at this time. To more deeply understand how the pandemic impacted anxiety in adolescents around the world, the current study adopted a qualitative approach to synthesising the global evidence on adolescents' lived experiences of anxiety during the pandemic.

**Methods:**

Five databases (Academic Search Complete, British Education Index, Education Research Information Centre, APA PsycINFO, and Scopus) were searched for studies that included qualitative data reported by adolescents on their lived experiences of anxiety during the pandemic. After duplicate records were removed, 348 records were title and abstract screened, a shortlist of 117 publications for full text screening, resulting in 34 papers to be included in the review.

**Results:**

Thematic analysis of data uncovered adolescents' experiences of anxiety during the pandemic in relation to a wide range of risk factors (i.e., academic stressors, family and economic stressors, social isolation, online dangers, uncertainties and health‐related concerns) and protective factors (e.g., social support, personal coping, accurate information and clear guidelines, digital tools) in different developmental contexts.

**Conclusions:**

These findings can guide the development of effective practices and policies for young people navigating the complexities of the post‐pandemic world.

## 1 Introduction

Adolescence is a critical inflection point for lifespan trajectories of mental health and wellbeing (Blakemore and Mills 2014). A critical aspect in the promotion of adolescent mental health is support across multiple domains, with peers, parents, and teachers playing a critical role in youth wellbeing. In the winter of 2020, the novel coronavirus began to spread, transforming the day‐to‐day lives of individuals across the globe in all domains. As many governments shut down and health policies were put in place to slow the spread of the virus, the lives of adolescents and their families were fundamentally altered as youth navigated school closures, structural and political instability, isolation, health concerns, and grief (Fegert et al. 2020; Guessoum et al. 2020). Previously identified protective factors were diminished and risk factors for anxiety increased (Magson et al. 2021; Zoellner et al. 2024). Concurrently, rates of anxiety increased among all groups of adolescents (Racine et al. 2021), even amongst groups who previously reported lower rates of anxiety.

Despite widespread interest in understanding how the pandemic affected adolescent mental health and emotion development, little work has been done to examine youth‐identified factors that affected anxiety during this time. Given the transformative effects of COVID‐19 and the subsequent instability in adolescents' daily lives, it is important to better understand adolescents' lived experiences of the pandemic and how protective and risk factors for anxiety amplified or attenuated pandemic‐related distress. A better understanding of these factors could provide future guidance for parents, educators, and policymakers and help adolescents better navigate periods of instability. The present study examines these relationships through a qualitative systematic review identifying themes across a diverse population of youth.

### 1.1 Anxiety in Adolescence

Anxiety disorders are the most prevalent mental health concern among adolescents (Rapee et al. 2019; Kessler et al. 2012; Merikangas et al. 2010). As adolescents navigate new developmental challenges in biological, psychological, and social spheres, they are increasingly vulnerable to the increases in anxiety symptoms at both clinical and subclinical levels (Spence and Rapee 2016). Although biological and genetic factors explain some vulnerability to anxiety (Rapee et al. 2023), for many youth the psychosocial changes that occur during adolescence can be particularly impactful, such as emotional reactivity, increased salience of peers and sensitivity to peer stress, and developmental stressors (Rapee et al. 2019; Spence and Rapee 2016; Forbes et al. 2019).

Importantly, stressors in social domains may not be innately risky and youth are expected to face new challenges in the transition from childhood to adolescence, as an ability to navigate developmentally appropriate challenges is an essential aspect of positive youth development (Masten and Monn 2015). Although exposure to stress helps youth master skills that help them later navigate challenges in adulthood, nonnormative stressors or a greater number of concurrent stressors can lead to increases in anxiety symptoms, as youth may be overextended in their ability to respond to challenging environments and have insufficient resources to cope (Masten et al. 2021). In particular, environmental factors such as parental stress (Griffith et al. 2023; Lei et al. 2023), family environment (Wong et al. 2024), and school climate (Aldridge and McChesney 2018) have been found to be concurrently and prospectively associated with anxiety symptoms.

### 1.2 The COVID‐19 Pandemic

As the novel coronavirus spread across countries, public health measures led to a shift to remote schooling and work at home accommodations for many adolescents and their families. Other measures, such as social distancing, restrictions around public gatherings, and requisite masking, were also employed to reduce the spread of the virus. Although responses to the pandemic varied by region, the transformed global environment required that adolescents navigate the normative changes of adolescence in historically novel environments. Adolescents’ usual psychosocial challenges were further intensified by higher levels of individual and community loss, illness, and increased exposure to traumatic events than the generations that came before (McMahon et al. 2023; Scott et al. 2021). Not only did the rates of known risk factors (e.g., loss, traumatic experiences) increase (Zhang et al. 2024), but the changes that occurred with the onset of the pandemic limited adolescents’ access to social supports that previously have been identified as buffers for anxiety symptoms in adolescence (Magson et al. 2021).

It is unsurprising that the disruptions that occurred globally during the pandemic have been linked to adolescent internalizing problems. Although mental health researchers identified worrisome increases in both depressive and anxiety symptoms in youth before the pandemic (Gunnell et al. 2018), rates of stress and anxiety increased at a greater rate than would otherwise have been expected during this time (Barendse et al. 2023; Racine et al. 2021). These increases are consistent with prior literature on youth mental health during events such as natural disasters, and regional and political conflict (Osokina et al. 2023; O'Neill and Rooney 2018).

### 1.3 The Current Study

Although existing research highlights the necessity of understanding increased rates of anxiety in adolescence during and after the pandemic, current work offers little insight into adolescents' own reported risk and protective factors to amplify or ameliorate their anxiety during the COVID‐19 pandemic. Understanding adolescents' experiences of anxiety and underlying mechanisms is crucial for supporting them and addressing their needs. To address this gap in the literature, the current study evaluated existing literature examining adolescents' lived experiences of anxiety during the COVID‐19 pandemic. Specifically, our study drew on data from 34 studies published between 2020 and 2024 that had data which provided information on youth's experiences of anxiety during the pandemic. In conducting a qualitative systematic review that identified themes of protection and risk during the pandemic, we sought to elucidate future areas for researchers to target for prevention and intervention efforts during times of instability.

## 2 Method

The review methods were informed by the Preferred Reporting Items for Systematic Reviews and Meta‐Analyses (PRISMA) checklist (Moher 2009). The review protocol was pre‐registered in PROSPERO (Ref: CRD42024522843).

### 2.1 Eligibility Criteria

This study aimed to review empirical (primary) research reporting first‐person qualitative experiences of adolescent anxiety during pandemic. Therefore, to be included in the review, studies had to meet the following inclusion criteria: (1) be an empirical study on adolescent anxiety, (2) have a sample of adolescents (10–18 years), (3) be conducted during the pandemic, (4) utilise qualitative methods. Mixed method studies were included if their qualitative aspects fit with the eligibility criteria.

Studies were excluded if: (1) adolescents were not the respondents, (2) the study did not examine anxiety in relation to the pandemic, (3) the sample included younger or older age groups, and the findings were reported across all age groups, (4) the study was not peer reviewed, (5) the study was not available in English.

### 2.2 Search Strategy

The search strategy was developed and iteratively refined by the research team, led by a senior academic (JS) with extensive experience in conducting systematic reviews in the field. To strengthen the strategy, we consulted existing systematic reviews on related topics to inform our construct mapping and development of search terms. Scoping searches of relevant keywords (e.g., adolescence, anxiety, pandemic) further informed the final strategy.

Between December 2023 and February 2024, five databases (Academic Search Complete, British Education Index, Education Research Information Centre, APA PsycINFO, and Scopus) were systematically searched to identify qualitative studies that focused on adolescents' lived experiences of anxiety during the COVID‐19 pandemic. These databases were selected to ensure good coverage of the literature on psychology, adolescent development, and education given that school closures were a major factor impacting adolescents during the pandemic.

#### 2.2.1 Search String

A comprehensive list of search terms and relevant synonyms across for constructs (i.e., anxiety, adolescence, COVID‐19 pandemic, qualitative method) were combined using Boolean operators ‘OR’ within each concept and “AND” between constructs. Table 1 presents the final search string. Searches were limited to title and abstract. Filters were applied to retrieve peer‐reviewed publications published in English. Backward citation searches were also completed to identify additional records. In cases where a record could not be accessed, the authors were emailed to ask for a copy.

**Table 1:** Final search string.

Construct	Synonyms
Anxiety	anxiety OR anxious OR internalizing OR internalising OR somatic OR phobia OR panic OR “mood disorder” OR agoraphobia
Adolescence	adolescen* OR “young person” OR “young people” OR youth OR teenage*
COVID‐19 pandemic	Covid‐19 OR covid OR coronavirus OR pandemic
Qualitative	qualitative OR inductive OR narrative OR discourse OR IPA OR phenomenology OR interview OR perception OR “lived experience” OR “grounded theory” OR “document analysis” OR “life history” OR naturalistic OR “thematic analysis” OR voice

### 2.3 Study Selection

Records were merged using an Excel spreadsheet and duplicates were removed. Remaining records were dual screened by the research team (BK, NS, KM, LB) at the title and abstract level to determine whether they met the eligibility criteria. The agreement between them was moderate (77.6%; Cohen's *K* = 0.51). Disagreements were resolved by involving another rater (JS) who made the final decision through discussions. The remaining records were, then, examined more closely through full‐text screening by individual raters (BK, NS, KM, LB). In cases of indecision on whether a record met inclusion criteria, bilateral discussions between all screeners were conducted and final decisions were made.

**Figure 1:**
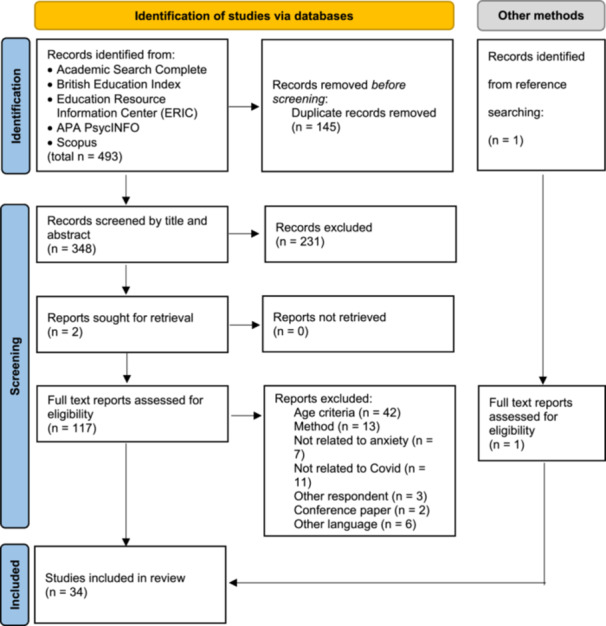
PRISMA diagram for the identification and screening of studies included in the review.

The database searches retrieved 493 records, with 145 duplicates (see Figure 1 for PRISMA diagram; Page et al. 2021). After the removal of duplicates, 348 records were screened and 231 of them were excluded at title and abstract level. Two records were not accessible, so they were retrieved by contacting their authors. A total of 117 records went through full‐text screening, with 84 of them being excluded at the end due to not fitting the inclusion criteria. With an additional record that met inclusion criteria identified through reference tracking, the total number of studies included in the review was 34.

### 2.4 Data Extraction

Key data were extracted by four authors (BK, NS, KM, LB) from each study into an Excel spreadsheet, relating to study characteristics (e.g., authors, publication year, location, sample size and demographics, study design, contextual details in relation to the pandemic), anxiety (type, definition), method (qualitative data collection tools), and analysis approach. Furthermore, findings (e.g., summary findings, themes, and participant quotes) related to adolescent's experiences of anxiety in relation to the pandemic were extracted from the studies for thematic analysis.

### 2.5 Quality Appraisal Approach

The quality of the included studies was assessed by four authors (BK, NS, KM, LB) using the Quality Appraisal Checklist for Quantitative, Qualitative, and Mixed Methods Studies (QQM; Symonds and Tang 2024). QQM includes a total of 14 indicators for qualitative studies in which the studies are scored across from 1 to 3 for indicator. The indicator scoring, then, are summed to calculate a final score for each study, with 14 as the lowest (quality) and 42 as the highest (quality) score. For qualitative studies, QQM provides cut‐off scores where a score of 21 and below indicates poor quality, scores between 22 and 33 indicate moderate quality, and a score of 34 and above indicates high quality. None of the studies were excluded based on the quality assessment. The cut‐off points were used as a guide to categorise studies based on their scores (e.g., moderate or high quality) to critically evaluate each study and its contribution to the overall synthesis.

### 2.6 Data Analysis

Thematic analysis with a reflexive lens (Braun and Clarke 2021) was employed to explore key risk and protective factors associated with adolescent anxiety during the pandemic. Analysis was performed via NVivo software. The analysis followed a structured process, where data were (1) familiarised though rereading, and (2) systematically coded to capture key features. This was followed by (3) grouping initial codes into broader themes, (4) reviewing and developing these themes to ensure they accurately represented the data, (5) defining and refining the themes to ensure analytic clarity, and (6) synthesising the themes into a coherent narrative that addresses the research aims.

This was a reflexive, organic, and iterative process, in which coding and theme development were shaped by engagement with the data and our interpretative lens. The primary analysis was led by two authors (NS, LB) who regularly met to share insights, discuss interpretations and reflect on their own positioning. Other members of the team provided critical feedback to support theme development and ensure the analytic narrative was coherent and grounded in the data set.

### 2.7 Collective Reflexivity Statement

Our research team comprised a diverse group of scholars, including both early career researchers and senior academics, with disciplinary backgrounds spanning psychology (including developmental and clinical psychology), education, social work, and mental health. While all researchers were based in Western academic institutions, the team brought a range of lived experiences and perspectives, including ethnic minority backgrounds, migrant experiences, and members for whom English is a second language. These intersecting identities informed our individual and collective interpretations of the data, our sensitivity to issues of equity and inclusion, and our attentiveness to cultural and contextual variation in how adolescent anxiety is experienced and reported.

This diversity also shaped our analytic process. Throughout the project, we engaged in critical reflection, both individually and through collaborative discussions, about how our own assumptions, disciplinary training, and sociocultural locations might influence what we attended to in the data and how we framed emerging themes. For example, researchers with professional experience in education and social work offered insights into how school‐based and structural factors may shape adolescent anxiety, while those with developmental psychology training brought a nuanced understanding of age‐related trajectories. The mix of academic seniority within the team further supported open dialogue and the surfacing of divergent interpretations, contributing to a richer and more layered analysis.

While we recognise that no analysis is free from the influence of researcher subjectivity, we see our positionality as a resource rather than a limitation. Our aim was not to eliminate bias, but to be transparent about how our perspectives shaped the interpretive work of thematic analysis.

## 3 Results

### 3.1 Overview of the Studies

Table 2 summaries the main characteristics of the studies included in the review. The studies were published between 2020 and 2023 and involved a total of 3227 adolescents. The majority of data came from countries in the Global North (*n* = 26 studies, reaching at least 2492 participants) including USA, UK, Canada, Singapore, and countries in Europe; with the remaining data being collected in the Global Majority countries of Bangladesh, Brazil, Côte d'Ivoire, Ethiopia, India, Lebanon, South Africa, Turkey (*n* = 8 studies, reaching 889 participants). Twenty‐four studies recruited from the general population, while nine involved samples with health related characteristics (e.g., obesity), neurodiversity, and/or a mental health diagnosis (including self‐diagnosis). Where reported or available, data suggested that 1131 participants identified as male, 1704 as female, and 1 as trans, nonbinary or gender questioning. Participants' ages ranged from 10 to 19 years. Where reported, participants were sampled from diverse ethnic/racial populations as well as from mixed income (D'amico et al. 2020; Lockyer et al. 2022; Pearcey et al. 2024; Rogers et al. 2021; Sandhu and Barn 2023; Scott et al. 2021; Scott et al. 2023; Şenkal et al. 2023; Stewart et al. 2023) or low income backgrounds (Banati et al. 2020; Carey et al. 2022; Duby et al. 2022; Nguyen‐Rodriguez et al. 2023; Stiles‐Shields et al. 2022, 2024).

**Table 2:** Main characteristics of the studies included in the review.

Study	Location	*N*	Gender	Age range; mean	Ethnicity/race	Study design	Data collection; analysis	Data collection period
Appel et al. (2023)	USA	13	4 Male 9 Female	11 to 18; N/R	33% White; 77% People of colour	Longitudinal; qualitative	Focus group; thematic analysis	February– November 2021
Bailie and Linden (2023)	UK (Northern Ireland)	17	12 Male 5 Female	10 to 14; 11.9	N/R	Cross‐sectional; qualitative	Semi‐structured interview; thematic analysis	Winter and Spring 2021
Banati et al. (2020)	Ethiopia, Côte d'Ivoire, Lebanon	568	213 Male 236 Female	10 to 19; N/R	N/R	Cross‐sectional; qualitative	Semi‐structured interview; thematic analysis	April– June 2020
Branquinho et al. (2020)	Portugal	N/A	N/A	16 to 18; N/A	N/A	Cross‐sectional; qualitative	Open‐ended questionnaire; content analysis	April– May 2020
Carey et al. (2022)*	Ireland	10	7 Male 3 Female	10 to 13; N/R	N/R	Cross‐sectional; qualitative	Semi‐structured interview; Interpretative phenomenological analysis	August 2020
Coetzee et al. (2022)*	South Africa	7	1 Male 6 Female	12 to 13; 12.4	71.4% Afrikaans as native language; 28.6% English as native language	Cross‐sectional; qualitative	Semi‐structured interview; thematic analysis	July–September 2020
Coyle et al. (2022)*	Portugal	10	5 Male 5 Female	16 to 18; N/R	N/R	Cross‐sectional; qualitative	Semi‐structured interview; thematic analysis	November 2020
D'amico et al. (2020)	USA	20	9 Male 11 Female	12 to 16; 14	95% American Indian/Alaska Native	Cross‐sectional; qualitative	Semi‐structured interview; Rapid assessment	May–July 2020
Davis and Stanton (2023)	USA	7	1 Male 5 Female 1T/NB/GQ	10 to 15; N/R	N/R	Longitudinal; mixed‐methods	Open‐ended questionnaire; multiphase phenomenological process	Summer 2020
Duby et al. (2022)	South Africa	20	0 Male 20 Female	15 to 19; N/R	N/R	Cross‐sectional; mixed‐methods	Semi‐structured interview; thematic analysis	November 2020–March 2021
Gadagnoto et al. (2022)	Brazil	22	5 Male 17 Female	14 to 18; 15.7	45.55% brown skin, 31.8% white, 18.2% black, 4.5% yellow	Cross‐sectional; qualitative	Open‐ended questionnaire, Social phenomenology	October– December 2020
Giannakopoulos et al. (2021)	Greece	9	N/R	12 to 17; N/R	N/R	Cross‐sectional; qualitative	Open‐ended questionnaire; Phenomenological‐hermeneutical approach	April 2020
Giannopoulou et al. (2022)	Greece	187	65 Male 122 Female	17 to 18; N/R	N/R	Cross‐sectional; mixed‐methods	Open‐ended questionnaire; thematic analysis	April–May 2021
Hughes and Jones (2024)	UK	9	4 Male 5 Female	12 to 16; N/R	N/R	Cross‐sectional; qualitative	Semi‐structured interview; thematic analysis	N/R
Lew‐Koralewicz (2022)	Poland	10	9 Male 1 Female	16 to 18; 16.7	N/R	Cross‐sectional; qualitative	Semi‐structured interview; interpretative phenomenological analysis	N/R
Lockyer et al. (2022)	UK	21	9 Male 12 Female	10 to 13; N/R	42.9% White British, 33.3% Pakistani, 19.1% other	Cross‐sectional; qualitative	Semi‐structured interview; thematic analysis	August–September 2020
Morsa et al. (2022)*	Canada (Quebec)	15	7 Male 8 Female	14 to 17; N/R	N/R	Cross‐sectional; qualitative	Focus group; thematic analysis	May 2020
Nguyen‐Rodriguez et al. (2023)*	USA	46	23 Male 23 Female	10 to 12; 11	91% Mexican	Cross‐sectional; qualitative	Focus group; thematic analysis	March–August 2020
Nilsson et al. (2021)	Sweden	702	296 Male 404 Female	15 to 19; N/R	N/R	Cross‐sectional; mixed‐methods	Open ended questionnaire; content analysis	July–November 2020
Parker et al. (2021)	USA	12	6 Male 6 Female	12 to 17; 15.17	100% Black	Cross‐sectional; qualitative	Interview; transcendental phenomenological studies	June–July 2020
Pearcey et al. (2024)	UK (Northern Ireland)	17	5 Male 12 Female	11 to 16; N/R	N/R	Cross‐sectional; qualitative	Semi‐structured interview; reflexive thematic analysis	December 2020–February 2021
Peterle et al. (2022)	Brazil	15	N/R	15 to 18; 16.03	N/R	Cross‐sectional; mixed‐methods	Open‐ended questionnaire; content analysis	April–July 2021
Rogers et al. (2021)	USA	407	203 Male 204 Female	14 to 17; 15.24	52% White, 20% African American, 17% Hispanic/Latinx, 3% Asian American, 1% American Indian or Alaska Native, 7% mixed/other backgrounds	Longitudinal; mixed‐methods	Open‐ended questionnaire; grounded theory and Thematic analysis	April 2020
Sandhu and Barn (2023)	India	24	N/R	15 to 17; N/R	N/R	Cross‐sectional; qualitative	In‐depth interviews and mapping exercise; thematic analysis	March 2020
Sarkadi et al. (2021)	Sweden	330	N/R	13 to 18; N/R	N/R	Cross‐sectional; qualitative	Open‐ended questionnaire; content analysis	N/R
Scott et al. (2021)	UK (England)	31	13 Male 18 Female	13 to 17; 14.59	94% White British	Longitudinal; mixed‐methods	Diary and Semi‐structured interview; thematic analysis	July – November 2020
Scott et al. (2023)	UK (England)	26	10 Male 16 Female	13 to 17; 14.46	100% White British	Longitudinal; qualitative	Diary and semi‐structured interview; case profiles	June 2020 – September 2021
Şenkal et al. (2023)*	Turkey	19	8 Male 11 Female	10 to 18; N/R	N/R	Cross‐sectional; Qualitative	Focus group; thematic analysis	2021
Sifat et al. (2022)	Bangladesh	60	30 Male 30 Female	13 to 15; N/R	N/R	Cross‐sectional; qualitative	In‐depth interviews; thematic analysis	2021
Soon et al. (2022)	Singapore	41	29 Male 12 Female	14 to 16; N/R	63% Chinese, 22% Malay, 7% Indian, %7 other backgrounds	Cross‐sectional; mixed‐methods	Semi‐structured interview; thematic analysis	October 2020 – March 2021
Stewart et al. (2023)	UK (Scotland)	518	187 Male 311 Female 8T/NB/GQ	14 to 18; 15.88	85% White, 6% Asian, 21% mixed, 2% African, 1% Arab, 1% Caribbean or Black, %1 unspecified	Cross‐sectional; mixed‐methods	Open‐ended questionnaire; thematic analysis	August – September 2020
Stiles‐Shields et al. (2022)	USA	17	3 Male 14 Female	12 to 17; 15.88	82.4% Black or African American, 5.9% American Indian or Alaska Native, 5.9% mixed background, 5.9% unspecified	Cross‐sectional; mixed‐methods	Focus group; thematic analysis	“early 2021”
Stiles‐Shields et al. (2024)	USA	17	3 Male 14 Female	12 to 17; 15.88	82.4% Black or African American, 5.9% American Indian or Alaska Native, 5.9% mixed background, 5.9% unspecified	Cross‐sectional; qualitative	Focus group; thematic analysis	“early 2021”
Vella Fondacaro et al. (2023)	Malta	N/A	N/A	12 to 18; N/A	N/R	Longitudinal; mixed‐methods	Open‐ended questionnaire	August 2020 – October 2022

*Note: T/NB/Q* = Trans, nonbinary, gender questioning. *N/R* = Not reported.

* Participant characteristics provided only for the age group (10–18 years) relevant to the current review.

The study designs were mostly cross‐sectional (*n* = 28), whereas several studies (*n* = 6) reported longitudinal approaches. Eleven studies reported mixed‐methods approaches, whilst the remainder (*n* = 23) utilised qualitative approaches only, such as semi‐structured interviews, focus groups, open‐ended questionnaires, and diaries. The qualitative data were analysed using various approaches such as thematic analysis, content analysis, interpretative phenomenological analysis, or various other phenomenological approaches. Fourteen studies explicitly stated that the data were collected during a national or regional lockdown (Bailie and Linden 2023; Branquinho et al. 2020; Carey et al. 2022; Coetzee et al. 2022; D'amico et al. 2020; Duby et al. 2022; Lockyer et al. 2022; Nguyen‐Rodriguez et al. 2023; Sandhu and Barn 2023; Scott et al. 2021; Scott et al. 2023; Şenkal et al. 2023; Sifat et al. 2022; Vella Fondacaro et al. 2023).

### 3.2 Quality Appraisal

The studies, in general, reflected moderate (*n* = 9) to high (*n* = 25) levels of quality, with total scores ranging between 25 and 40 (see Table 3; see Supporting Material for all scores). A general shortcoming across many studies, but particularly among the studies that received moderate quality rating, was researchers' failure to recognize their own positionality and biases in the research process (Banati et al. 2020; Branquinho et al. 2020; Coetzee et al. 2022; Coyle et al. 2022; D'amico et al. 2020; Davis and Stanton 2023; Duby et al. 2022; Giannakopoulos et al. 2021; Giannopoulou et al. 2022; Hughes and Jones 2024; Lockyer et al. 2022; Morsa et al. 2022; Nilsson et al. 2021; Peterle et al. 2022; Rogers et al. 2021; Sarkadi et al. 2021; Şenkal et al. 2023; Sifat et al. 2022; Stewart et al. 2023; Stiles‐Shields et al. 2022, 2024; Vella Fondacaro et al. 2023). Furthermore, more than half of the studies failed to report a justification for the sample size in relation to the planned analysis method (Appel et al. 2023; Banati et al. 2020; Branquinho et al. 2020; Coetzee et al. 2022; Coyle et al. 2022; D'amico et al. 2020; Davis and Stanton 2023; Giannopoulou et al. 2022; Hughes and Jones 2024; Pearcey et al. 2024; Sandhu and Barn 2023; Sarkadi et al. 2021; Scott et al. 2023; Sifat et al. 2022; Stewart et al. 2023; Stiles‐Shields et al. 2022, 2024; Vella Fondacaro et al. 2023). Similarly, the majority of the studies failed to report the number of people who did not consent to participate (Appel et al. 2023; Bailie and Linden 2023; Banati et al. 2020; Branquinho et al. 2020; D'amico et al. 2020; Davis and Stanton 2023; Giannakopoulos et al. 2021; Giannopoulou et al. 2022; Morsa et al. 2022; Nguyen‐Rodriguez et al. 2023; Nilsson et al. 2021; Sandhu and Barn 2023; Sarkadi et al. 2021; Scott et al. 2023; Sifat et al. 2022; Soon et al. 2022; Stiles‐Shields et al. 2022, 2024).

**Table 3:** Quality appraisal scores of studies using the QMM (Symonds and Tang 2024).

Study	Total
Appel et al. (2023)	34
Bailie and Linden (2023)	39
Banati et al. (2020)	27
Branquinho et al. (2020)	30
Carey et al. (2022)	39
Coetzee et al. (2022)	36
Coyle et al. (2022)	29
D'amico et al. (2020)	35
Davis and Stanton (2023)	33
Duby et al. (2022)	34
Gadagnoto et al. (2022)	40
Giannakopoulos et al. (2021)	32
Giannopoulou et al. (2022)	31
Hughes and Jones (2024)	34
Lew‐Koralewicz (2022)	37
Lockyer et al. (2022)	38
Morsa et al. (2022)	37
Nguyen‐Rodriguez et al. (2023)	40
Nilsson et al. (2021)	34
Parker et al. (2021)	37
Pearcey et al. (2024)	37
Peterle et al. (2022)	37
Rogers et al. (2021)	38
Sandhu and Barn (2023)	33
Sarkadi et al. (2021)	31
Scott et al. (2021)	39
Scott et al. (2023)	37
Şenkal et al. (2023)	35
Sifat et al. (2022)	25
Soon et al. (2022)	37
Stewart et al. (2023)	36
Stiles‐Shields et al. (2022)	36
Stiles‐Shields et al. (2024)	36
Vella Fondacaro et al. (2023)	37

### 3.3 Results of the Thematic Analysis

The extracted study results were organized into themes using a mixture of deductive and inductive approaches. First, each statement was coded into two major categories of risk and protective factors. Second, the researchers analysed the data within the risk/protective factors categories using an inductive approach where the segments of data were organized into codes that reflected the meaning (symbolic and/or semantic) of the risk/protective factor identified. The approach was discussed at several points in the process, and the researchers agreed that the data fitted well into the overarching themes. Below, we report on the sub‐themes within the two overarching themes of protective factors that facilitated better adjustment and reduced anxiety and risk factors that were perceived as amplifying difficulties.

#### 3.3.1 Protective Factors

##### 3.3.1.1 Social Support

Having a support network in place alleviated anxious feelings by reducing loneliness and providing a sense of stable engaged relationships with significant figures in their lives (e.g., educators, family members, peers). Adolescents felt grateful for being able to talk to a parent or carer about how they were feeling and coping, as one 15‐year‐old teen said: “My relationship with my parents and other relatives seems much better. We're all talking much more” (Rogers et al. 2021, p. 47). Some also felt having their siblings around helped them cope with lockdowns, suggesting that their absence would have led them to “feel alone” (Pearcey et al. 2024, p. 17). For many adolescents, being able to talk to friends and keep in contact was described as valuable. Participants noted that there were topics that they would more openly discuss with friends rather than anyone else in their lives, as seen in this 15‐year‐old teen's words: “I talk about different things with my friends than I would with my parents. It is being able to discuss stuff with my friends that's, it's a good way to, like, stay mentally calm” (Pearcey et al. 2024, p. 18).

In school, adolescents noticed that adults were actively trying to be more flexible with their expectations, using different ways to communicate, and showing more understanding about schoolwork: “They were a little lenient and they gave us more time if we needed it. And if something happened all we had to do was email them and they would give us more time.” (Parker et al. 2021, p. 309). They also appreciated efforts made to maintain the relationship and monitor the effects the pandemic had on their mental health. They emphasized that having teachers who made active efforts to inquire about their well‐being and offer support made a difference in how they were feeling. This 14‐year‐old female noted her teacher's active attempts to help students: “She was really putting a lotta emphasis on helping the ones that really did not know how to cope with being at home.” (Stiles‐Shields et al. 2024, p. 148).

##### 3.3.1.2 Personal Coping

As traditional support systems like schools, extracurricular activities, and face‐to‐face social interactions were restricted, adolescents described their ability to harness different coping mechanisms to reduce their anxiety including using different outlets such as keeping diaries, and maintaining hobbies (e.g., painting, listening to music) and routines. They reported establishing time‐ and activity‐management strategies, such as structuring their days around routines of physical activities, schoolwork, and virtual meetings with friends. One study found that boys appeared to benefit more from recreational activities and digital connectivity, which buffered against emotional distress (Stewart et al. 2023).

These actions provided adolescents with a sense of normalcy and control, in a rapidly changing environment, as can be seen in this 14‐year‐old teen's comment: “Most helpful thing to me is being able to read and play computer games and play guitar. Just carry on with my hobbies which I was doing before lockdown.” (Pearcey et al. 2024, p. 11). Additionally, several adolescents identified religious faith as a salient psychological resource for reducing anxiety, as shown by one teen's words: “Prayer helps me stay calm… just sit in there and talk, that calmed my anxiety because I was talking to God” (Parker et al. 2021, p. 308).

##### 3.3.1.3 Accurate Information and Clear Guidelines

In places where governments responded early (e.g., the Netherlands, Seattle, Greece) with science‐led messaging and targeted restrictions, adolescents expressed anxiety about infection and, at times, imposed stricter confinement on themselves than was mandated (Appel et al. 2023; Giannakopoulos et al. 2021). Adolescents’ narratives indicated that access to accurate pandemic‐related information increased their trust in the authorities and the community, which in turn helped reduce their anxiety by fostering a perception that the pandemic was manageable and progressing towards a solution, as exemplified in these teens’ accounts: “We have taken precautions… and that reassures me…”, “Both doctors and experts do the best they can to help people feel safe.” (Giannakopoulos et al. 2021, p. 6).

##### 3.3.1.4 Digital Tools

Besides providing a platform to meet friends during lockdowns, adolescents described spending time on social media to distract their attention from the overwhelming, anxiety‐provoking news regarding the spread of the pandemic. Besides offering a temporary relief, apps and digital tools also helped adolescents build coping skills, as highlighted by this 16‐year‐old:

I think YouTube gives me ideas of what I should do. How to calm myself down. Sometimes, you can even put a video where they tell you like, “Okay. You count down from this number to this number, or you breathe in and you breathe out.” I feel that helps for me a lot, when I'm stressing or feeling anxious for some particular things.

(Stiles‐Shields et al. 2022, p.8)

Lastly, for some adolescents, particularly those with prior mental health difficulties or neurodevelopmental conditions, the shift away from school environments led to reductions in stress and social pressure (Lew‐Koralewicz 2022). They perceived the shift to virtual learning and socializing as an opportunity to express themselves more authentically, as it helped reduce their anxiety about how they were perceived by their peers. This is illustrated in these teens' words: “Not having to go to school has boosted my self‐confidence as I no longer had to worry what people thought of me”, “I have been able to be myself, not my depressing school self” (Stewart et al. 2023, p.251). However, many also faced intensified symptoms due to disrupted routines and diminished access to support services (Pearcey et al. 2024).

#### 3.3.2 Risk Factors

##### 3.3.2.1 National Restrictions and Pandemic Stages

National responses to the COVID‐19 pandemic varied in timing, intensity, enforcement mechanisms, and socio‐political context. These variations were associated with adolescents' anxiety in several ways. In countries that implemented strict lockdowns (e.g., Ireland, Greece, Singapore), sometimes enforced by police or military (e.g., South Africa), Adolescents reported experiencing anxiety due to the loss of personal freedom and fear of being caught if they did not comply with social isolation rules (Bailie and Linden 2023; Coetzee et al. 2022). When such restrictions lasted for extended periods, especially, but not exclusively, in developing countries (e.g., Ethiopia, Lebanon, India, Côte d'Ivoire), they exacerbated anxieties over educational gaps and financial strain, as seen in this teen's words: “My family started skipping many of our needs to be able to pay the rent and the internet fees. We are skipping a lot of food due to the increasing prices. We have a lot of fights and problems…” (Banati et al. 2020, p. 1627). In contrast, in countries with more lax or decentralized approaches (e.g., Sweden), adolescents reported less anxiety over disruptions to daily life and instead expressed greater concern about becoming infected or infecting others (Nilsson et al. 2021; Sarkadi et al. 2021).

Some adolescents initially viewed lockdown as a welcome break from academic and social pressures, describing it as a “holiday” (Pearcey et al. 2024, p. 15). However, when restrictions continued, many adolescents' anxieties shifted from infection risk to long‐term disruptions in education and social life (Pearcey et al. 2024; Scott et al. 2023), with a gradual return to pre‐pandemic stressors such as bullying and community violence (Coetzee et al. 2022), as manifested in this Mexican American teen's words: “Sometimes I don't feel safe because my mom likes to watch news and then sometimes like there's shooting down my area. It makes me feel not safe.” (Nguyen‐Rodriguez et al. 2023, p.17).

##### 3.3.2.2 Academic Stressors

Adolescents described experiencing both internal and external sources of anxiety related to the transition to online learning. They voiced struggles finding internal motivation to study and carry on with schoolwork, especially when facing multiple distractions at home, lacking structure, and dealing with constant changes in their academic curriculum. As was noted by this teen: “Schools are different, and I have struggled a bit with this, I feel as though some of the guidelines contradict each other and this worries me” (Stewart et al. 2023, p. 253).

The shift to virtual schooling also underscored disparities in access to resources, including reliable internet and appropriate study environments (e.g., quiet, private). This inequity in access and remote learning experiences led to frustration among some students, with some reporting they were worried about falling behind academically: “My friend can attend the online classes. But there is no notice of [socioeconomic] classes in our school. I feel upset sometimes. I am lagging behind.” (Sifat et al. 2022, p. 6). “I stopped studying because we don't have internet, we don't have money to pay for it… The connectivity is very bad and the electricity always cuts, which make it impossible to attend the classes” (Banati et al. 2020, p. 1626).

Teens disclosed being reluctant to share their difficulties when others were present online, as emphasized by one student reporting that he “wouldn't ask questions online” (Soon et al. 2022, p. 76). The disruptions in their learning environment, combined with academic struggles, gave rise to performance anxiety that manifested in lower confidence in their capabilities and future career prospects. This was especially prominent in the descriptions of adolescents undergoing transitions (e.g., approaching graduation), who feared that their educational paths would be disrupted by the constant changes in education systems and decreased academic achievements, as explained by this teen:

More nervous to return to school when they open again, I know that I'm not the only one! I think a lot of us are scared, we are still unsure about our GCSEs. The government hasn't really been that clear on how they are dealing with our futures, typical!

(Scott et al. 2023, p. 7)

Additionally, some adolescents indicated the pressure to succeed in a new and different learning environment and reduced support from teachers further intensified their anxiety: As this teen explained: “This education method has caused us severe stress and depression. We are given more lessons and homework than we usually get at school without explaining them to us and … with little support from our teachers.” (Banati et al. 2020, p. 1626).

##### 3.3.2.3 Family and Economic Stressors—More Is Less?

While some adolescents emphasized the opportunities gained to interact with family members, others described the downside of these changes. Living in close quarters with multiple family members for extended periods sometimes led to increased conflict and stress within households, exacerbating feelings of anxiety. As this teen describes: “I spent time with my mom a lot before, now we're both so stressed and agitated that it's putting a strain on our relationship.” (Rogers et al. 2021, p. 46). Adolescents emphasized the importance of having time for themselves as a valuable resource to cope with stressful interactions with family members: “To actually get private time relaxing in my room. Everyone is home so there's always noise and someone knocking at my door.” (Rogers et al. 2021, p. 46).

Socioeconomic status (SES) played a critical role in shaping adolescent anxiety during the pandemic. Youth from low‐income households reported heightened stress due to economic instability, food insecurity, and limited access to digital education platforms (Duby et al. 2022; Sifat et al. 2022). In Lebanon, adolescents described having to choose between buying food and purchasing masks, illustrating the compounded nature of financial hardship and infection anxiety (Banati et al. 2020). In high‐income countries like the UK, adolescents in disadvantaged areas still experienced more severe lockdown measures and associated stress due to higher local infection rates and household overcrowding: “…Well, I wouldn't be able to go full day without hearing how many deaths”, “It's just when you'd go out when you would go out you'd see the army on the street which is give you the shivers” (Carey et al. 2022, p. 185). Financial instabilities also heightened tensions at home, adding anxieties over limited resources and income uncertainties. One teen said:

My father has lost his job. My brother used to earn money by tutoring. But he lost tuition owing to the pandemic situation. We do not have any income source now, and I am afraid of how we are going to survive.

(Sifat et al. 2022, p. 6)

##### 3.3.2.4 Social Isolation

The isolation caused by lockdowns and social distancing measures disrupted many adolescents’ daily routines (e.g., eating, sleeping, physical activities, and socializing). With fewer opportunities for in‐person interactions, adolescents expressed having fewer opportunities to distract themselves from ruminating or worrying about the pandemic or the future. They also expressed becoming more worried about losing touch with their friends over time. This led to feelings of loneliness, fear of being excluded, and concerns about peer rejection, as highlighted by this teen: “I wish I could've seen my friends because they're the people I'm most close to so being away from them for many months was really upsetting and difficult for me” (Stewart et al. 2023, p. 253).

Several studies noted that these anxieties were intensified among girls, who reported greater emotional distress and somatic symptoms of anxiety, such as headaches and tremors (Peterle et al. 2022; Branquinho et al. 2020). In Ethiopia, Lebanon, and Côte d'Ivoire, girls' limited access to digital technologies, due to restrictive gender norms, further exacerbated feelings of isolation and stress. As one female adolescent expressed: “The tension has increased dramatically… We are all at home suffering from a bad psychological state” (Banati et al. 2020, p. 1627).

##### 3.3.2.5 Online Dangers

To overcome their loneliness and isolation, many adolescents turned to social media in search of social connection. However, the pressure to keep an online presence combined with exposure to the curated, at times idealized depictions of others' lives intensified adolescents' anxieties over inadequacy. One teen expressed her apprehension: “If the smartphone is what is giving this person anxiety and depression because of the people on social media, they could not wanna be on their phone.” (Stiles‐Shields et al. 2022, p. 10).

##### 3.3.2.6 Uncertainties and Health‐Related Concerns

Concerns about contracting the virus or about the health and safety of loved ones were constant sources of adolescents' anxiety. As expressed in this teen's words: “I'm partly worried that my loved ones will get sick. My older relatives and parents mean an incredible amount to me, and I am of course worried that they will fall ill or even lose their life to Corona.” (Sarkadi et al. 2021, p.943).

When governments displayed fragmented or poorly coordinated responses, such as in Brazil or Bangladesh, where political tensions between federal and local governments were prominent (Ahmed et al. 2024; Touchton et al. 2021), adolescents struggled to make sense of the conflicting information surrounding the pandemic, which further heightened fears and anxieties. One teen described being concerned about being exposed to inaccurate information online:

My parents try to explain to me what's going on, I talk to my mom, or read the news on the Internet, but reading doesn't help, on the contrary, it makes my thinking even more chaotic. It's hard to put it all in order. There are a lot of contradictions and unknowns. The information is inconsistent, and you can get lost in it all and there are no clear guidelines because it all changes and you have to adapt all the time.

(Lew‐Koralewicz 2022, pp. 5‐6).

Finally, several adolescents expressed feeling anxious due to the macro‐impact of the pandemic and its unknown long‐term effects: “I am concerned about the overload that may affect the health care system if people do not take responsibility and limit their contacts and how the health care system and mainly its staff are affected by long‐term stress.” (Sarkadi et al. 2021, p. 944). “I worry about what's gonna happen to our economy” (Rogers et al. 2021, p. 47).

## 4 Discussion

Adolescence is a period of significant psycho‐social development, marked by emotional reactivity, brain plasticity, and heightened vulnerability to environmental influences (Rapee et al. 2019). From a developmental resilience perspective (Masten 2014), when adolescents are exposed to stressors, but are able to manage them, it can build their resilience and competency (Masten and Monn 2015). However, when stressors cumulate, they can deplete individuals' resources and place them at risk of developing psychopathology (Cicchetti 2013; Ellis et al. 2011). The sudden upheaval caused by the COVID‐19 pandemic exacerbated existing challenges adolescents were facing, while also introducing new sources of anxiety along several life domains. Several studies documented a rise in adolescents' anxiety during the pandemic (Magson et al. 2021; Racine et al. 2021), while others found anxiety levels decreased or remained stable (Hollenstein et al. 2021; Li et al. 2022).

Adolescents' experience of pandemic‐related anxiety may have been shaped by their personal coping skills, family dynamics, economic stability, developmental stage, and their social and cultural environments. This study provided a synthesized literature review of 34 qualitative studies exploring adolescentss lived experience of anxiety during the COVID‐19 pandemic. Included studies offered insights quantitative studies may have overlooked—such as giving voice to stressors that were not necessarily apparent in surveys and factors identified by the adolescents themselves as protective against the negative impact of the pandemic. Hence, the review provides a broad understanding of factors that might contribute to a reduced sense of anxiety and can inform clinicians and policymakers on possible intervention targets to help prevent the development of psychosocial difficulties.

### 4.1 The Context Matters

Government responses to the COVID‐19 pandemic appeared to influence adolescent experiences in comparable ways. Similar to the typology of parenting styles (Maccoby 1992), when governments adopted an authoritative approach, providing coherent information, engaging in clear communication with the public, and setting well‐defined rules, adolescents tended to be more trusting and made autonomous choices to protect themselves and others from infection. These actions contributed to reduced anxiety.

In contrast, when governments were either authoritarian, imposing strict, punitive measures with limited explanation, or neglectful of the difficulties civilians were facing, adolescents reported increased anxiety. This included uncertainty about the future and heightened fears regarding potential impacts on both them and broader systems such as healthcare and the economy.

Anxiety levels were not static but evolved with the pandemic timeline. Initial lockdowns were sometimes perceived positively, particularly by adolescents who experienced a reprieve from academic or social stressors (Gadagnoto et al. 2022; Pearcey et al. 2024). This is consistent with previous findings indicating some youth showed improved well‐being in early lockdowns due to decreased school‐related stress (Ellis et al. 2020). However, as the pandemic persisted, concerns shifted toward educational disruption, uncertainty about the future, and social isolation, mirroring longitudinal findings who noted that adolescent mental health declined as the pandemic dragged on.

In addition, the pandemic appeared to exacerbate structural inequities in adolescents’ access to support systems and coping resources, with those who were already vulnerable being severely affected. Adolescent girls reported increased social restrictions and caregiving responsibilities (e.g., in Ethiopia and Lebanon), and youth from lower‐income households described heightened stressors related to food insecurity, household overcrowding, and limited access to educational resources. Findings highlight how economic pressures may have compounded stress and disrupted developmental and academic trajectories. These findings align with prior research indicating that being a female, financial hardship, and digital exclusion were significant predictors of mental health deterioration during the pandemic (Deng et al. 2023; Gassman‐Pines et al. 2020; Halldorsdottir et al. 2021; Magson et al. 2021).

Although quantitative studies have reported small to moderate increases in pandemic‐related mental health challenges, when comparing younger and older adolescents (Madigan et al. 2023; Reiss et al. 2024), or adolescents with pre‐existing mental or physical health conditions to those without (Hawes et al. 2022; Masi et al. 2021), our review suggests that these factors may not determine the valence of anxiety so much as its focus. Older adolescents appeared more prone to future‐oriented anxieties (e.g., concerns about education, employment, and long‐term uncertainty), while younger teens more often described immediate disruptions to daily routines. Similarly, pre‐existing mental health conditions had heterogeneous effects: for some, the reduced demands of in‐person social interactions offered temporary relief from school‐related stressors; for others, the same isolation amplified feelings of loneliness and cut off access to vital peer support networks.

Importantly, the qualitative nature of the studies reviewed may have afforded a more nuanced understanding of these patterns, highlighting the complexity of adolescents lived experiences and amplifying the voices of vulnerable youth who may be underrepresented or oversimplified in large‐scale, survey‐based research.

### 4.2 Social Networks—A Double‐Edged Sword

Amid social isolation and related restrictions, adolescents described their social networks as having the potential to be both valuable and harmful for them. Consistent with previous findings (Ellis et al. 2020; Magson et al. 2021; Qi et al. 2020), when significant people in their environment, including parents, educators and friends, were being supportive, curious of how they were doing, and offering help or guidance, adolescents felt they were better able to address their anxieties. However, when adolescents' home environment was characterized by frequents conflicts, when individuals in their social networks showed little interest in them, or were overwhelmed by their own anxieties and insecurities, adolescents reported feeling lonely and more anxious. This finding suggests that it was not merely the quantity of social relations but rather their quality that contributed to adolescents' well‐being. From an attachment perspective, having warm, sensitive close relations with their parents, facilitates adaptation by increasing adolescents' ability to connect and form close, supportive relationships with their peers (Scharf et al. 2004). Additionally, having teachers who continuously pay attention to students' needs, listen to their concerns and guide them on making choices – can cultivate competence and resilience (Noddings 2019). In contrast, when adolescents' relationships are characterized by conflict or disregard of their emotions and needs, it can result in difficulties relying on others for help and ill‐being (Scharf 2007). Taken together, supportive social relations are protective resources in the face of stressful life events (Bonanno et al. 2007). Notably, these experiences of conflict or support were likely mediated by familial and community resources, such as student‐to‐teacher ratios and parental financial stressors that may have made it more difficult for adolescents to receive support from parents and teachers (Dändliker et al. 2022).

Furthermore, adolescents emphasized using social media to mitigate feelings of isolation, maintain social bonds and gain access to mental health support resources. Some adolescents (primarily those with previous mental health difficulties) described the switch to virtual learning and socializing as an opportunity to express themselves in a more authentic manner as their anxiety regarding how they were perceived by their peers were reduced. Nevertheless, for others, the pressure to keep an online presence combined with exposure to the curated, at times idealized depictions of others' lives intensified anxieties over inadequacy. The shift to virtual schooling also underscored disparities in access to resources, including reliable internet and appropriate study environments (e.g., quiet, private). Prior findings both before and during the pandemic, presented mixed results on the effects of social media use on adolescents' development (Marciano et al. 2022; Odgers et al. 2020). The conflicting results may be related to teen's motivations for using social media and the content to which they are exposed. When social media is used as a resource to connect to others, share experiences and provide distractions from overwhelming stressors, it can alleviate negative feelings (Popat and Tarrant 2023). Nevertheless, at a developmental period characterized by heightened sensitivity to peer approval and acceptance (Somerville 2013), social media use can also exacerbate social comparison, thereby increasing fears and ruminations over being left out or rejected by peers, resulting in greater anxiety and ill‐being (Hayran and Anik 2021; Liu et al. 2021; Vall‐Roqué et al. 2021).

### 4.3 Continuity in a Changing World

Echoing previous findings (Lessard and Puhl 2021), as schools closed, extracurricular activities and face‐to‐face social interactions were restricted, some adolescents struggled finding internal motivation to learn and persist with schoolwork, especially when facing multiple distractions at home, lacking structure and facing constant changes in their academic curriculum. Others portrayed personal agency and an ability to harness different coping mechanism to reduce their anxiety—these included establishing time‐ and activity‐management strategies, such as structuring their days around routines of physical activities, schoolwork, keeping diaries and virtual meetings with friends. Under stressful conditions, routines have been shown to contribute to a sense of predictability, and controllability and provide an outlet to express personal identity (Hou et al. 2019). From a drive to thrive framework (Hou et al. 2018), the pandemic created challenges for adolescents to sustain their daily routines (e.g., sleep time, eating and exercising behaviours), those who were able to continue to invest and engage in life tasks, even in the face of severe stress, had better chances of preserving their mental health.

### 4.4 Transitions and Uncertainties

Adolescents undergo significant changes in several life domains, including puberty, school transitions, new relationships, and growing autonomy and independence. These developmental transitions can encompass uncertainty, and lead to anxiety over one's competencies and future prospects (Grills‐Taquechel et al. 2010). In line with previous findings, pandemic‐related uncertainties, such as fear of contracting the disease or infecting others, inadequate pandemic‐related information, parental job loss, and disruptions in school curriculum, were causes for additional stress at an already sensitive developmental period (Brooks et al. 2020; Buzzi et al. 2020; Low and Mounts 2022). Our review noted that for older adolescents approaching graduation, inconsistent schooling created greater fears that their educational and future career paths would be deflected.

The ability to tolerate uncertainty involves openness to new experiences and new meanings, and decision making in ambiguous conditions (Koerner and Dugas 2008). It may require coping skills, such as planning and anticipating outcomes, that are still developing during adolescence (Bailen et al. 2019). This might explain why adolescents expressed feeling overwhelmed by the disruptions in their lives and the pressure to succeed in a new and rapidly changing environment. However, situational characteristics and not merely personal ones may have contributed to adolescents' well‐being. Whereas before the pandemic, focusing on their futures, may have alleviated anxieties over the developmental transitions by fostering more goal directed behaviour (Yang et al. 2021), the ever‐changing regulations and lack of schooling availability may have reduced adolescents’ opportunities to plan their near and long‐term future, thereby leaving them anxious and worrisome over what is to come.

From a developmental cumulative risk perspective (Ellis et al. 2009; Evans et al. 2013), the combination of unpredictability (constant changes in schooling, spread of the pandemic), social threats (peer rejection, family conflicts) and deprivation (loss of economic resources, and loss of mental and educational support stemming from school closures) placed adolescents at increased risk to suffer from feelings of anxiety. However, even under these conditions, adolescents who were able to identify and make use of personal and social resources in their environment, described coping and adaptation. Although the conditions of the pandemic were certainly unique, many of the coping and adaptation strategies were not. Practitioners and educators may help adolescents navigate future uncertainty or difficulties by working with youth to identify what strategies and resources they found helpful during the pandemic and utilizing these protective factors to address post‐pandemic challenges.

### 4.5 Practice and Policy Implications

This literature review presented a synthesis of existing evidence of adolescents' lived experience of anxiety during the COVID‐19 pandemic. Findings highlight the need to address adolescents' emotional responses to the pandemic and to pay attention to their own voices when considering when and where to intervene. Consistent with other studies, this study indicated that during a global health crisis, adolescents held complex perceptions of different factors in their lives as having the potential to be both helpful and stress inducing (e.g., spending more time with family members or on social media). Adolescents felt anxiety over changes in their academic structures, the availability of support figures, and the need to adjust to a rapidly changing environment. However, when given opportunities to experiment with new or improved capabilities, when maintaining continuity in routines and social relations and when feeling supported and seen by important others in their lives—adolescents were able to feel less anxious.

Building on these insights, creating supportive environments that address the multifaceted nature of adolescent anxiety should be prioritised. For practitioners, this means adopting holistic approaches that consider the interplay of academic pressures, family and economic challenges, and social isolation, while actively promoting protective factors such as strengthening social support networks and enhancing young people's personal coping skills. Educators can play a critical role by fostering safe and inclusive school climates that reduce academic stress and social isolation, while also integrating clear communication strategies and accurate information to help adolescents navigate uncertainties related to health and pandemic‐related changes.

Policymakers, meanwhile, are encouraged to develop flexible policies that recognise the diverse developmental contexts of young people and the ongoing impacts of the pandemic. This includes ensuring access to digital tools that facilitate connection and mental health support, alongside investing in community resources that support families facing economic and social stressors. By embedding these considerations into practice and policy, stakeholders can more effectively respond to adolescent anxiety in ways that are sensitive to evolving challenges and supportive of young people's resilience and wellbeing in the post‐pandemic landscape.

### 4.6 Limitations

This review has several limitations. First, some of the studies included had only moderate methodological quality (e.g., lack of adequate information on sample or recruitment and retention), which merits caution in interpretating the findings. Second, some of the studies had extremely small samples, while others lacked diversity (e.g., including mostly females or high‐income families) making generalizability difficult. Third, only studies published in English were included, which may have biased our findings. Last, as most included studies were cross‐sectional, some issues raised by participants may have been the cause of pre‐pandemic difficulties. Nevertheless, as we were interested in the individualized experience of adolescents during the pandemic, all samples provide important insights into adolescents' lived experience during a world health crisis.

The findings of this review must be considered in light of the timing of data collection across included studies. While the pandemic is often referred to as a singular event, it encompassed a series of evolving phases, such as initial lockdowns, gradual reopenings, periods of heightened transmission, and the rollout of vaccines, each with distinct implications for adolescents' experiences and mental health. Most of the studies in this review collected data during 2020 and 2021, a period largely characterised by widespread uncertainty, school disruptions, and shifting public health measures. Although the specific timing and nature of restrictions varied by country, the common thread across studies was the instability and unpredictability of daily life during this period, which may have contributed to elevated levels of anxiety among adolescents. Where available, we noted whether studies reported lockdowns or school closures at the time of data collection. However, due to inconsistencies in reporting and differences in national timelines, we were not able to categorise all studies according to specific pandemic phases. Nonetheless, the overarching climate of disruption and uncertainty helps explain and interpret the risk and protective factors identified.

### 4.7 Conclusion

The COVID‐19 pandemic placed a high emotional toll on adolescents. Some were better able to adjust to the rapid changes and accommodate uncertainties in their lives, while others struggled. Future studies on anxiety or emotional responses to a macro‐level crisis should continue to probe adolescents' experiences as they differ from those of adults and children. Gathering adolescents' accounts of their struggles and coping mechanisms can help inform mental health professionals on resilience factors that contribute to adolescents' adjustment and on targets for interventions. This review highlights the need to create opportunities for adolescents to take responsibility over their routines, while also providing structure, scaffolding, and constant, sensitive support, to prevent detrimental consequences brought about by the crisis in global health.

## Author Contributions

**Buket Kara:** conceptualisation, methodology, investigation, data curation, formal analysis, visualization, writing – original draft, writing – review and editing. **Nitzan Scharf:** conceptualisation, methodology, investigation, data curation, formal analysis, writing – original draft, writing – review and editing. **Kathleen C McCormick:** conceptualisation, methodology, investigation, data curation, writing – original draft, writing – review and editing. **Linda Bhreathnach:** data curation, formal analysis, writing – original draft, writing – review and editing. **Candace Currie:** conceptualisation, methodology, supervision, writing – review and editing. **Jennifer Symonds:** conceptualisation, methodology, supervision, writing – original draft, writing – review and editing.

## Ethics Statement

Ethical approval was not required for this study as it is a systematic review of previously published literature and does not involve the collection of primary data. All included studies had obtained appropriate ethical approval as reported by their original authors.

## Conflicts of Interest

The authors declare no conflicts of interest.

## Supporting information

Supplementary Material_SLR adolescent anxiety.

## Data Availability

All data used in this systematic review are publicly available in the original published sources, which are cited within the manuscript.
