# Supplementary material for: Adolescent Anxiety During the COVID‐19 Pandemic: A Qualitative Systematic Review of Risk and Protective Factors
Source: J Adolesc. 2025 Aug 20;97(8):2048–66. doi: 10.1002/jad.70038 (PMC12682246; doi:10.1002/jad.70038)
Supplement: Supplementary file 1 — Supplementary Material_SLR adolescent anxiety. [file JAD-97-2048-s001.docx]

# Supplementary Material 1

Quality Appraisal of Studies using the QMM (Symonds & Tang, 2024)

| **Study** | **Setting** | **Recruitment** | **Administration** | **Consent** | **Ethics** | **Sample** | **Achieved sample** | **Sample size (justification)** | **Instrument content** | **Instrument rationale** | **Instrument example** | **Analysis method** | **Analysis example** | **Reflexivity** | **Total** |
| --- | --- | --- | --- | --- | --- | --- | --- | --- | --- | --- | --- | --- | --- | --- | --- |
| Appel et al. (2023) | 2 | 3 | 3 | 3 | 3 | 2 | 1 | 1 | 3 | 3 | 3 | 2 | 3 | 2 | 34 |
| Bailie & Linden (2023) | 3 | 3 | 3 | 3 | 3 | 3 | 1 | 2 | 3 | 3 | 3 | 3 | 3 | 3 | 39 |
| Banati et al. (2020) | 3 | 3 | 3 | 1 | 3 | 2 | 1 | 1 | 3 | 2 | 1 | 2 | 1 | 1 | 27 |
| Branquinho et al. (2020) | 2 | 3 | 3 | 3 | 3 | 3 | 1 | 1 | 3 | 2 | 1 | 2 | 2 | 1 | 30 |
| Carey et al. (2022) | 3 | 3 | 3 | 3 | 3 | 2 | 3 | 3 | 3 | 2 | 2 | 3 | 3 | 3 | 39 |
| Coetzee et al. (2022) | 3 | 3 | 3 | 3 | 3 | 3 | 3 | 1 | 3 | 2 | 2 | 3 | 3 | 1 | 36 |
| Coyle et al. (2022) | 2 | 2 | 2 | 1 | 1 | 2 | 2 | 1 | 3 | 3 | 3 | 3 | 3 | 1 | 29 |
| D'Amico et al. (2020) | 3 | 3 | 3 | 3 | 3 | 3 | 1 | 1 | 3 | 2 | 3 | 3 | 3 | 1 | 35 |
| Davis & Stanton (2023) | 3 | 3 | 3 | 3 | 3 | 2 | 1 | 1 | 3 | 2 | 2 | 3 | 3 | 1 | 33 |
| Duby et al. (2022) | 3 | 3 | 3 | 3 | 3 | 3 | 2 | 3 | 3 | 2 | 1 | 2 | 2 | 1 | 34 |
| Gadagnoto et al. (2022) | 3 | 3 | 3 | 3 | 3 | 3 | 3 | 2 | 3 | 3 | 3 | 3 | 3 | 2 | 40 |
| Giannakopoulos et al. (2021) | 3 | 1 | 3 | 2 | 1 | 2 | 1 | 3 | 3 | 3 | 3 | 3 | 3 | 1 | 32 |
| Giannopoulou et al. (2022) | 2 | 2 | 3 | 3 | 3 | 2 | 1 | 1 | 3 | 2 | 3 | 2 | 3 | 1 | 31 |
| Hughes & Jones (2024) | 3 | 3 | 3 | 3 | 3 | 3 | 3 | 1 | 3 | 2 | 1 | 3 | 2 | 1 | 34 |
| Lew-Koralewicz (2022) | 2 | 1 | 3 | 3 | 3 | 3 | 3 | 2 | 3 | 3 | 3 | 3 | 3 | 2 | 37 |
| Lockyer et al. (2022) | 3 | 3 | 3 | 3 | 3 | 3 | 3 | 3 | 2 | 3 | 2 | 3 | 3 | 1 | 38 |
| Morsa et al. (2022) | 3 | 3 | 3 | 3 | 3 | 3 | 1 | 2 | 3 | 3 | 3 | 3 | 3 | 1 | 37 |
| Nguyen-Rodriguez et al. (2023) | 3 | 3 | 3 | 3 | 3 | 3 | 1 | 3 | 3 | 3 | 3 | 3 | 3 | 3 | 40 |
| Nilsson et al. (2021) | 2 | 3 | 3 | 1 | 3 | 3 | 1 | 2 | 3 | 3 | 3 | 3 | 3 | 1 | 34 |
| Parker et al. (2021) | 1 | 3 | 3 | 2 | 3 | 3 | 2 | 2 | 3 | 3 | 3 | 3 | 3 | 3 | 37 |
| Pearcey et al. (2024) | 2 | 3 | 3 | 3 | 3 | 3 | 2 | 1 | 3 | 3 | 3 | 3 | 3 | 2 | 37 |
| Peterle et al. (2022) | 3 | 3 | 3 | 3 | 3 | 2 | 3 | 3 | 2 | 3 | 3 | 2 | 3 | 1 | 37 |
| Rogers et al. (2021) | 2 | 3 | 3 | 3 | 3 | 3 | 2 | 3 | 3 | 3 | 3 | 3 | 3 | 1 | 38 |
| Sandhu & Barn (2023) | 3 | 3 | 3 | 3 | 3 | 2 | 1 | 1 | 3 | 3 | 2 | 3 | 1 | 2 | 33 |
| Sarkadi et al. (2021) | 3 | 3 | 3 | 1 | 2 | 2 | 1 | 1 | 3 | 2 | 3 | 3 | 3 | 1 | 31 |
| Scott et al. (2021) | 3 | 3 | 3 | 3 | 3 | 3 | 3 | 3 | 2 | 3 | 2 | 3 | 3 | 2 | 39 |
| Scott et al. (2023) | 3 | 3 | 3 | 3 | 3 | 3 | 1 | 1 | 3 | 3 | 3 | 3 | 3 | 2 | 37 |
| Şenkal et al. (2023) | 3 | 2 | 3 | 3 | 2 | 2 | 2 | 3 | 3 | 3 | 2 | 3 | 3 | 1 | 35 |
| Sifat et al. (2022) | 3 | 1 | 2 | 3 | 2 | 2 | 1 | 1 | 2 | 2 | 2 | 2 | 1 | 1 | 25 |
| Soon et al. (2022) | 3 | 3 | 3 | 3 | 2 | 2 | 1 | 3 | 3 | 3 | 3 | 3 | 3 | 2 | 37 |
| Stewart et al. (2023) | 3 | 3 | 3 | 1 | 3 | 3 | 3 | 1 | 3 | 3 | 3 | 3 | 3 | 1 | 36 |
| Stiles-Shields et al. (2022) | 3 | 3 | 3 | 3 | 3 | 3 | 1 | 1 | 3 | 3 | 3 | 3 | 3 | 1 | 36 |
| Stiles-Shields et al. (2024) | 3 | 3 | 3 | 3 | 3 | 3 | 1 | 1 | 3 | 3 | 3 | 3 | 3 | 1 | 36 |
| Vella Fondacaro et al. (2023) | 3 | 3 | 3 | 3 | 3 | 2 | 3 | 1 | 3 | 3 | 3 | 3 | 3 | 1 | 37 |
